# Ethanol Extracted from Radix of Actinidia Chinensis Inhibits Human Colon Tumor Through Inhibiting Notch-signaling Pathway

**DOI:** 10.7150/jca.51275

**Published:** 2021-01-01

**Authors:** Wanle Hu, Chenchen Wu, Chenchen Yuan, Minyuan Chen, Chun Jin, Chenguo Zheng

**Affiliations:** Department of Coloproctology, The Second Affiliated Hospital and Yuying Children's Hospital of Wenzhou Medical University, No 109 Xueyuan Western Road, Wenzhou, Zhejiang Province, 325027, P.R. China.

**Keywords:** Colorectal cancer, EERAC, Cell viability, Cell migration, Notch-signaling pathway

## Abstract

**Background:** Colorectal cancer (CRC) is one of the most common tumors, and its five-year survival is still very low despite of the advance of treatment strategies. The antitumor effect of ethanol extracted from radix of Actinidia chinensis (EERAC) were identified in human colon cancer cells, but the underlying mechanism remains unclear.

**Methods:** Cell proliferation, migration, and invasion were measured with cell counting kit-8 (CCK-8), wound healing, and transwell assays. Cell apoptosis and cycle were detected by flow cytometry. Western blotting and qRT-PCR were used to measure expression of target molecules. Xenograft tumor assay was applied to detect the influence of EERAC on tumor growth.

**Results:** we found that EERAC inhibited the cell viability, migration, and invasion of SW480 cells in a concentration dependent manner, but promoted apoptosis and the cell percentage in S phase significantly. The suppression of notch-signaling pathway molecules, Notch1, Jagged1, and c-Myc, by EERAC was confirmed using western blotting and immunohistochemical staining. The significant inhibition of tumor growth by EERAC was also observed. Meanwhile, EERAC remarkably reversed the effects of mastermind like transcriptional coactivator 1 (MAML1, activator of notch-signaling pathway) on cell survival of SW480.

**Conclusions:** EERAC might be a promising chemotherapeutic agent for CRC treatment.

## Introduction

Colorectal cancer (CRC) is a common public health problem, being one of the most common gastrointestinal tumors in the world 1, 2. The bad prognosis induced tumor metastases and invasion leads to the low five-year survival rate of CRC patients 3. Although chemotherapy is accepted as standard treatment, large scale of patients suffers from the severe side effects, and drug resistance is commonly observed after long-term treatment. These adverse effects have greatly limited its clinical application 4. Therefore, in order to improve CRC survival rates, searching for a better therapeutic agent with enhanced activity is imminent.

Several Traditional Chinese Medicines isolated from natural plants have been widely used to treat tumors. For example, taxol and docetaxel were used to treat breast cancer and ovarian cancer, and irinotecan was applied for advanced CRC treatment 5-7. Several types of Traditional Chinese Medicines have been proved to be a potent anti-tumor agent 8. Flavonoids in Ageratum conyzoides and Chinese herbal formulas Miao-Yi-Ai-Tang have presented significant inhibition on cervical carcinoma and lung cancer, respectively 9.

Actinidia chinensis is an important type of raw materials in the fields of Traditional Chinese Medicines. Actinidia chinensis has presented promising therapeutic effect on several types of cancers including breast, liver, and gastric cancer 10, 11. The antitumor effects of radix Actinidia chinensis is that its root contains a large amount of terpenes, including ursolic acid, oleanolic acid and their derivatives 12-14. Besides, many studies have proved that ethanol extract from radix of Actinidia chinensis (EERAC) has an obviously curative effect on several types of tumors including hepatocellular carcinoma and lung cancer, 15-17. However, the specific regulatory mechanism remains unknown.

Activation of Notch-signaling pathway was related with angiogenesis, cell migration, invasion, differentiation, and differentiation 18, 19. Notch-signaling pathway contains notch receptors, notch ligands, and some key downstream proteins 20, 21. Notch 1, Jagged1, and c-Myc are the main notch receptor, notch ligand, and downstream molecule, respectively. Our previous study indicated that Jagged1 and Notch1 played a vital role during the prognosis, recurrence, metastasis of CRC 22. Therefore, notch-signaling pathway could regulate the occurrence, development, and prognosis of CRC. However, whether the EERAC can inhibit the proliferation and metastasis of CRC by down-regulating the Notch signal pathway has not been clearly described.

In the present study, we demonstrated that EERAC could potently suppress the CRC via down-regulating notch-signaling pathway *in vivo* and vitro. This is the first report implying the inhibition role of EERAC on CRC. Our results may provide new thought about the therapeutic method of CRC. Meanwhile, this research suggests that EERAC may have a good application value.

## Methods

### Reagents

The roots of Actinidia chinensis were purchased from Wenzhou Hospital of Integrated Traditional Chinese and Western Medicine. The EERAC (ethanol extract from radix of Actinidia chinensis) was isolated in our laboratory according to extraction technology of effective anti-cancer active ingredient of Actinidia chinensis root from Institute of Chemistry, Chinese Academy of Sciences (Patent publication No.: CN1977869A). The main active chemical components of EERAC were triterpene saponins, which identified by Libermannn Burchard reaction and foam test. Phosphate buffer saline (PBS), Fetal bovine serum (FBS), and tryspin were purchased from KeyGEN Biotech (Nanjing, Jiangsu, China).

### Cell culture

Colon cancer cell lines SW480 were obtained from Chinese Academy of Science (Beijing, China). Cells were cultured in DMEM medium (Invitrogen, Carlsbad, California) containing 5% FBS (Invitrogen, Carlsbad, California), and cultivated in the incubator at 37°C with 5% CO_2_. After passages, cells were incubated with ERRAC, and applied for different experiments.

### Drug preparation

EERAC was dissolved to the concentration of 400 μg/mL with 0.1% DMSO for stock (DMSO administration concentration <0.1%). The stock was diluted to 50, 100, 150, 200 μg/mL, respectively. A blank control group (PBS buffer) and a solvent control group (DMSO<0.1%) were used.

### CCK-8 assay

Cells (2×10^3^ cells/well) were maintained in 96-well plates. For cell viability assessment, transfected cells were incubated for 48 h. After treatment, CCK-8 reagent (Nanjing Jiancheng, Nanjing, China) was added, and OD at 450 nm was detected. The experiment was repeated 3 times.

### Transwell assay

The transwell chamber without matrigel (Keygen, Nanjing, China) was used for cell invasion experiment. Cells (1×10^6^) suspended with 200 μL medium were seeded in the top chamber. The bottom chamber was supplemented with 500 μL medium containing 5% FBS. After 24 h incubation, cells on the lower chamber were fixed using 4% polyoxymethylene for 15 min. Then, 0.1% crystal violet was used to stain cells for 20 min. Invasive Cells were calculated by capturing 3 fields using an inverted microscope (BX53, Olympus, Tokyo, Japan) at 400× magnification.

### Wound healing assay

The horizontal lines were drawn evenly on the back of the 6-well plate with ruler and marker pen. The interval between each two lines is 0.5-1.0 cm and the lines crossed the holes. Each well was seeded approximately 5×10^5^ cells and incubated overnight. Using a 100 μL pipette tip made scratches in the six-well plate. After scratching, the cell status at 0h was recorded by taking photos. Remove the original cultured medium and wash cells twice with 1 mL PBS. The prepared drug was added to the plate. Cells were cultured on the condition of 37°C and 5% CO_2_. Cells were recorded after 48 h by taking pictures.

### Western blot

SW480 cells were firstly treated with EERAC for 48 h. Then, cells were collected and lysed using lysis buffer (KeyGEN, Nanjing, China). Same amount of protein was loaded for 12% SDS-PAGE. Then, the gels were transferred to a PVDF membrane (Nanjing Jiancheng, China) electrophoretically. 5% non-fat milk was used for blocking. After 2 h of blocking, membrane was incubated with primary antibodies at 4°C for 12 h. After washing twice, secondary antibodies (1:2000) were applied for incubation for 4 h. TBST washing buffer was used to remove secondary antibodies, and Image J software was used to analyze protein bands. The primary antibodies used were listed as follows: Notch1 (1:800, #194123, Abcam, Cambridge, UK), Jagged1 (1:800, #109536, Abcam, Cambridge, UK), c-Myc (1:1000, #32072, Abcam, Cambridge, UK); beta-actin (1:1500,#16891, Abcam, Cambridge, UK).

### qRT-PCR

RNA was isolated using trizol reagent (TaKaRa, Beijing China). cDNA from different groups were measured by real time PCR with ChamQ^TM^ SYBR^®^ qPCR Master Mix (Vazyme, California, USA). The information of primers was listed as follows: Jagged1 (F: CGAGTCCTTTACGTGCGTCT, R: CAGACACACCGGTAGCCATT); Notch1 (F: GAGGCTTGAGATGCTCCCAG, R: ATTCTTACATGGTGTGCTGAGG); c-Myc (F: GAGGAGGAACGAGCTAAAAC, R: TGCTTGGACGGACAGGATG); GAPDH (F: ATGGGGAAGGTGAAGGTCG, R: TCGGGGTCATTGATGGCAACAATA). GADPH was used as internal control. 2^-ΔΔCT^ method was used to analyze the change of target gene expression.

### Flow cytometry

Cells (4 ×10^5^) were plated and cultivated in an incubator. After different treatments, cells were digested, and centrifuged to get cell pellet. Then, cells were suspended using 700 µl binding buffer containing 10 μl propidium iodide (Sigma, St. Louis, Missouri, USA) and 10 μl Annexin V-FITC (Life Technologies, Carlsbad, California, China). After incubation for 30 min in dark, apoptosis was detected using flow cytometric.

### Immunohistochemical staining

3% formalin was used for tissue fixation. After 24 h, tissues were embedded using OCT (Sigma, US). Tissues were sectioned in 10-μm thickness. Heating for 5 min using microwave for antigen repair, and then tissues were washed with PBS. Blocking was applied using goat serum. Then, primary antibody (1:1000) was applied to incubate tissues overnight, and secondary antibody was used to incubate sections for 3 h. DAB reagent was applied to culture tissues, and sections were analyzed with Olympus BX41 microscope (Tokyo, Japan).

### Xenograft tumor assay

The xenograft tumor assay was approved by the Institutional Animal Care of the Second Affiliated Hospital and Yuying Children's Hospital of Wenzhou Medical University (2019-066). Male nude mice (C57BL/6) were purchased from GemPharmatech (Nanjing, China), and randomly divided into different groups (3 mice/group). HT29 cells (2× 10^5^, 0.1mL) were injected subcutaneously into the back of mice. Animals were fed with EERAC (200 mg/kg) or sterile PBS. All mice were sacrificed after 5 weeks, and tumor weights were analyzed.

### Statistical analysis

The data were shown as mean ±SD, and analyzed with SPSS software (22.0, IBM, Armonk, USA). *t*-test was applied to compare the data of two groups. *p* <0.05 was believed statistically difference.

## Results

### EERAC significantly inhibited the proliferation, migration, and invasion of SW480 cells

**Figure 1:**
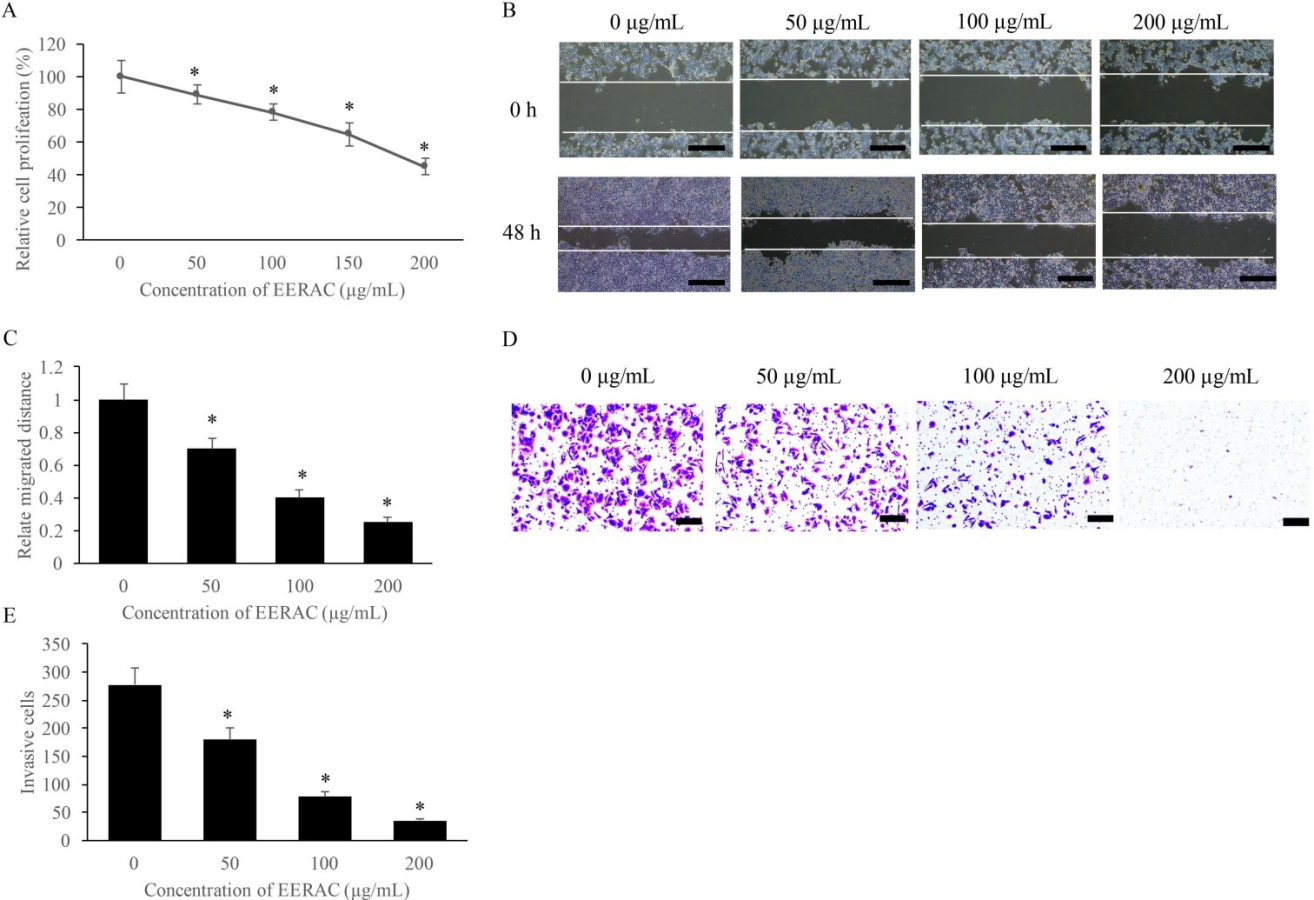
** EERAC significantly inhibited the proliferation, migration, and invasion of SW480 cells. (A)** Cell viability of SW480 cells treated with different concentrations of EERAC; **(B)** Representative images of wound healing assay after treatment with different concentrations of EERAC (Scale bar=500 µm); **(C)** Analysis of cell migration after treatment with different concentrations of EERAC; **(D)** Representative images of transwell assay after treatment with different concentrations of EERAC (Scale bar=200 µm); **(E)** Analysis of cell invasion after treatment with different concentrations of EERAC. *P <0.05 versus untreated EERAC (0 µg/mL).

The influence of EERAC on SW480 cells growth was measured with CCK-8, wound healing, and transwell methods. We found that EERAC suppressed the proliferation of SW480 cells on a dose-dependent mode (Figure 1A). Meanwhile, the migration and invasion of SW480 cells were also suppressed remarkably after treatment with various concentrations of EERAC (50-200 μg/mL) (Figure 1B-E). Therefore, EERAC might present potential ability of anti-colorectal cancer cells.

### EERAC remarkably increased the apoptosis rate of SW480 cells, and increased the cells percentage of S phase

**Figure 2:**
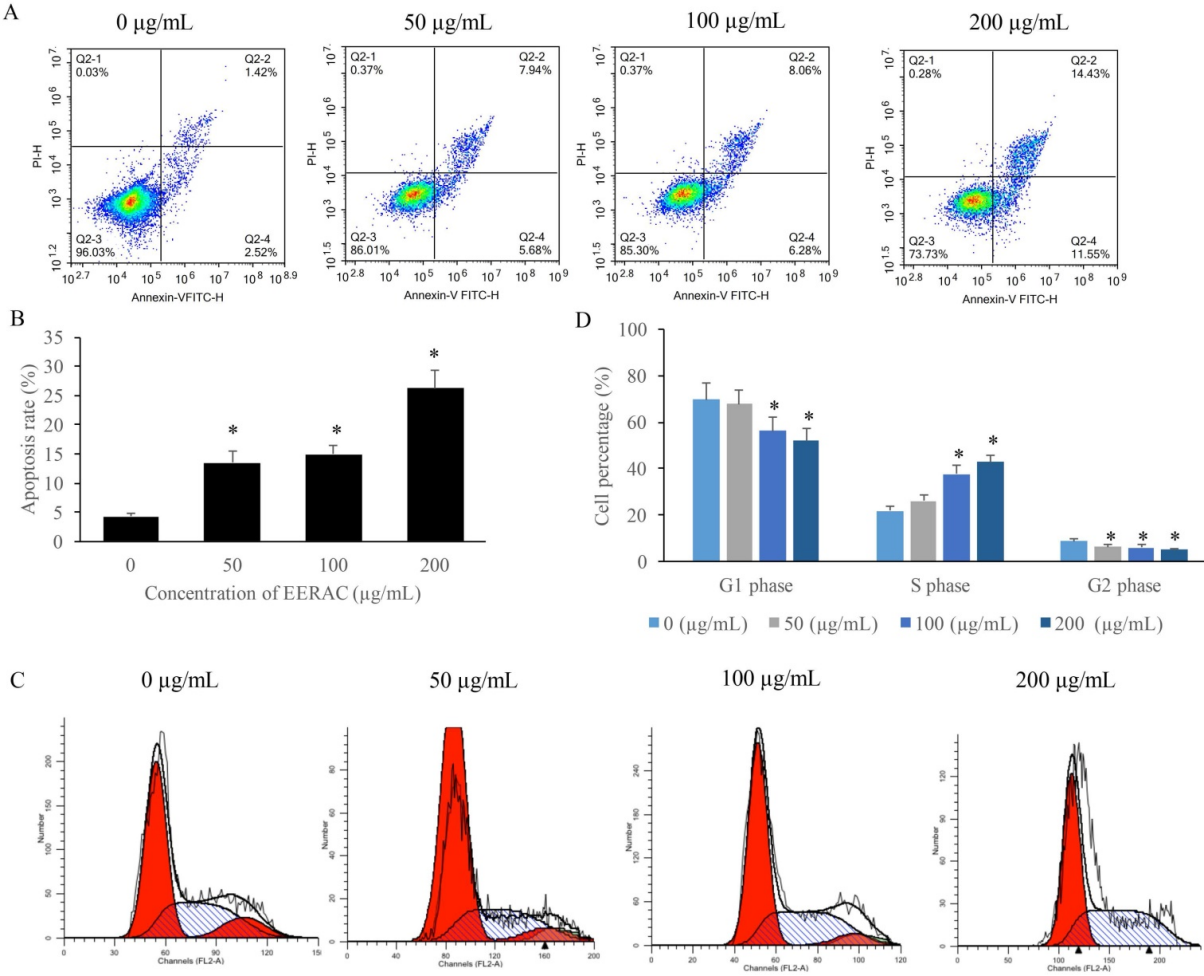
** EERAC remarkably promoted the apoptosis rate of SW480 cells, and increased the cells percentage of S phase. (A)** Cell apoptosis of SW480 cells was measured after treatment with EERAC; **(B)** Analysis of cell apoptosis after treatment with EERAC; **(C)** Cell cycle of SW480 cells was measured after treatment with EERAC; **(D)** Analysis of cell cycle after treatment with EERAC. *P <0.05 versus untreated EERAC (0 µg/mL).

We found that the apoptosis of SW480 cells was markedly increased after treatment with EERAC (Figure 2A-B), and the promotion of apoptosis was dose-dependent manner. Meanwhile, results of cell cycle indicated that cell percentage in the S phase were significantly increased by EERAC, but the cells in the G2 and G1 phases were increased (Figure 2C-D). These findings indicated that treatment with EERAC induced remarkable S phase arrest of SW480 cells.

### EERAC significantly inhibited the Notch signaling pathway

**Figure 3:**
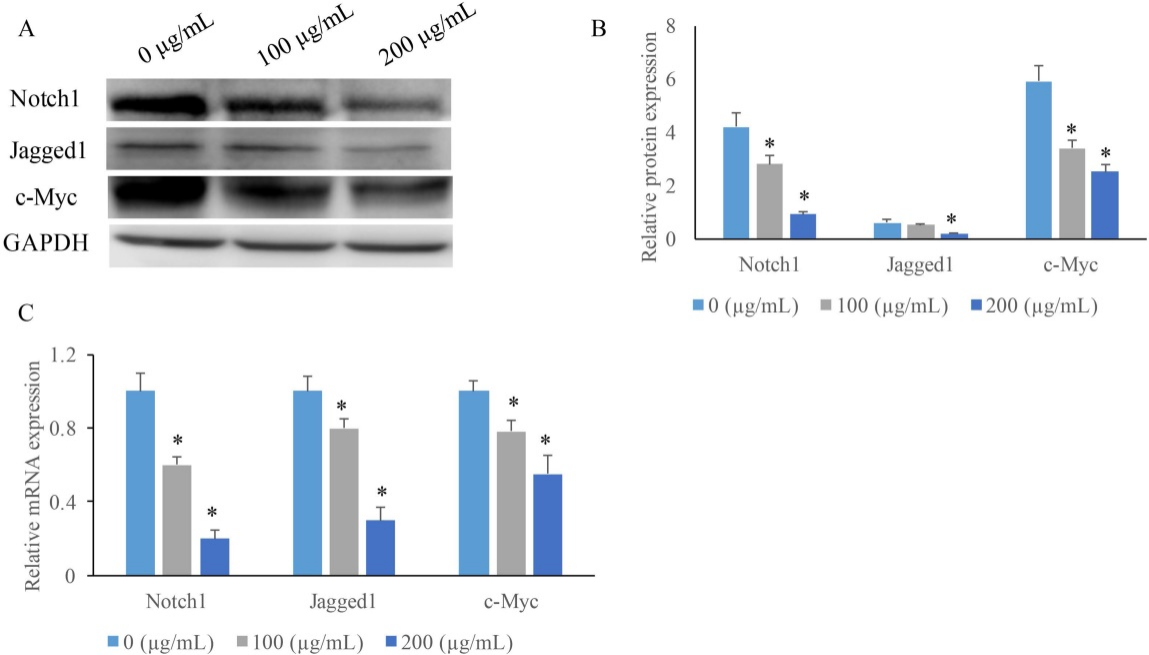
** EERAC significantly inhibited the notch-signaling pathway. (A)** Measurement of protein levels of c-Myc, Jagged1, and Notch1 by western blotting; **(B)** Analysis of protein expression of c-Myc, Jagged1, and Notch1; **(C)** Measurement of mRNA expression of c-Myc, Jagged1, and Notch1 by qRT-PCR. *P <0.05 versus untreated EERAC (0 µg/mL).

To investigate the potential mechanism how EERAC affects SW480 cells, we measured the expression of some key molecules in Notch signaling pathway after treatment with various concentrations of EERAC. We found that both the protein and mRNA expression of c-Myc, Jagged1, and Notch1 were suppressed by EERAC (Figure 3A-C). High concentration of EERAC (200 μg/mL) presented a stronger inhibition effect on Notch signaling pathway.

### EERAC remarkably inhibited the xenograft tumor *in vivo*

**Figure 4:**
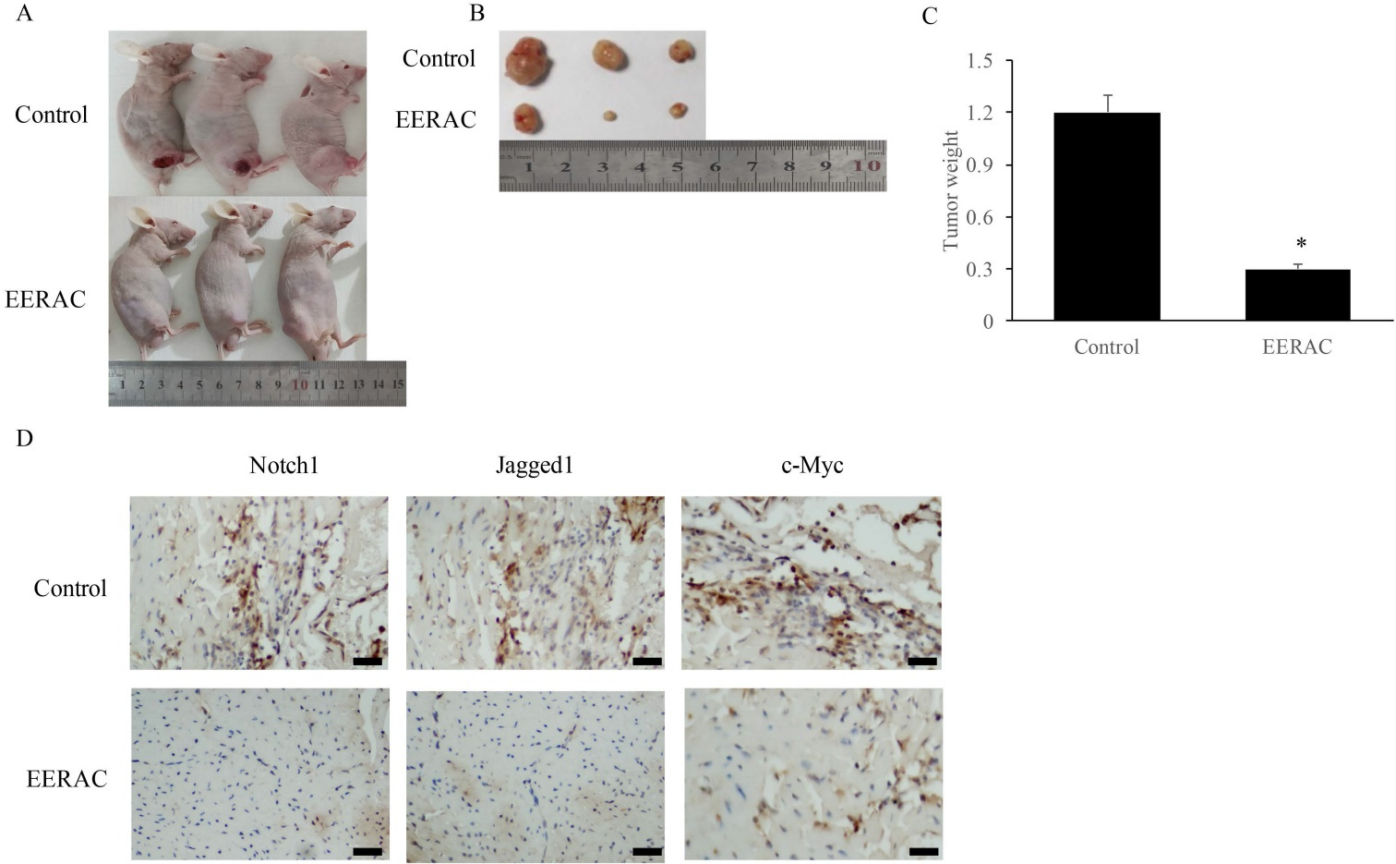
** EERAC remarkably inhibited the xenograft tumor *in vivo*. (A)** Morphology of nude mice operated with xenograft tumor (N=3 for each group); **(B)** Tumors were collected after sacrificing mice; **(C)** Analysis of tumor weight; **(D)** Detection of Notch1, Jagged1, and c-Myc in the tissues by IHC staining (Scale bar=500 µm). *P <0.05 versus control group.

After xenograft tumor, no mice death was observed after 3 weeks. We found that EERAC treatment significantly suppressed the tumor weight (Figure 4A-C). Meanwhile, we detected the levels of c-Myc, Jagged1, and Notch1 in the tumor tissues by IHC staining. The levels of c-Myc, Jagged1, and Notch1 were markedly suppressed by EERAC (Figure 4D), which was similar to the findings *in vitro*.

### EERAC remarkably reversed the influence of MAML1 on the survival of SW480 cells *in vitro*

**Figure 5:**
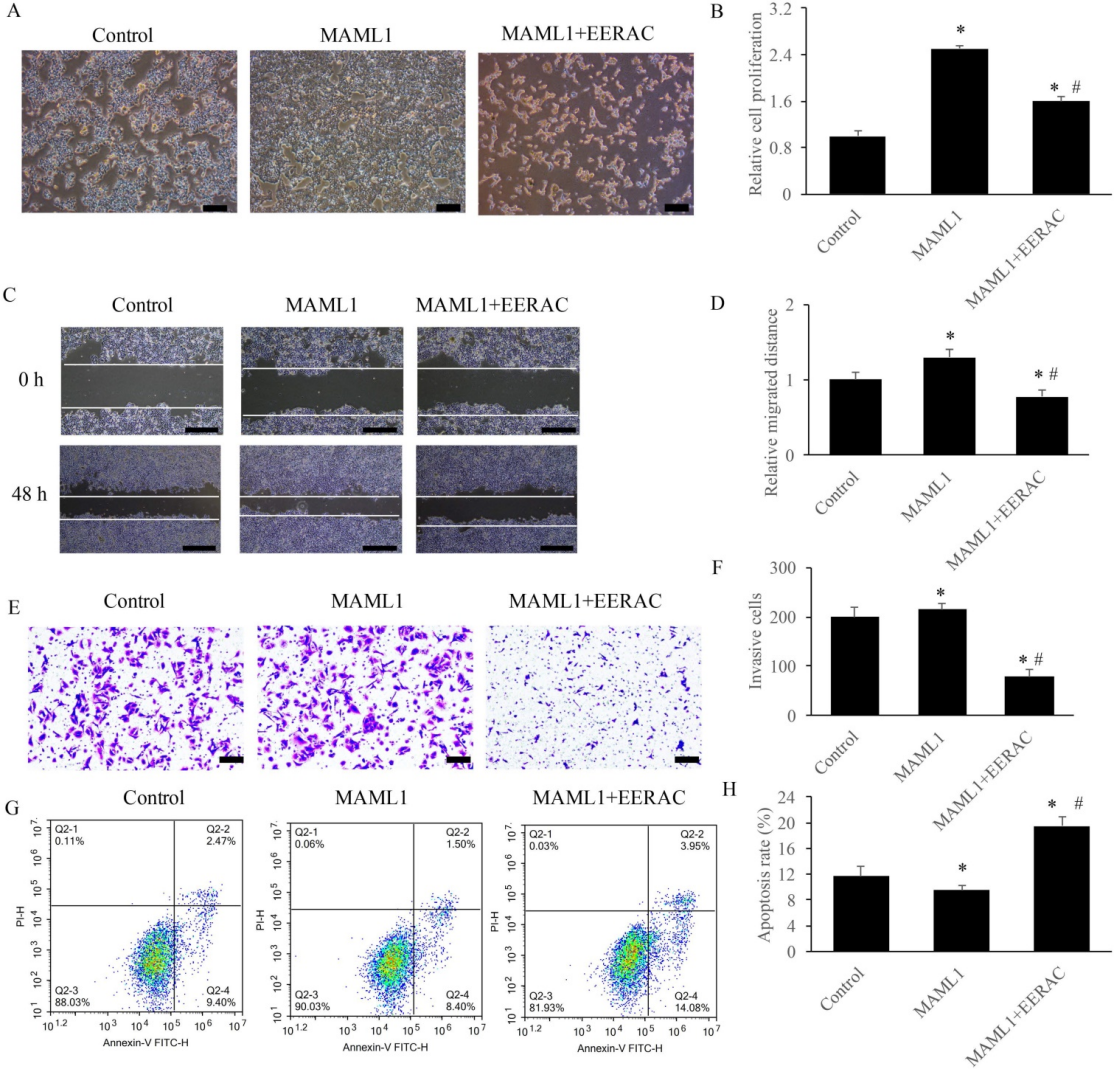
** EERAC remarkably reversed the influence of MAML1 on the survival of SW480 cells *in vitro*. (A)** Influence of MAML1 and EERAC on cell morphology (Scale bar=200 µm); **(B)** Influence of MAML1 and EERAC on cell proliferation; **(C)** Influence of MAML1 and EERAC on cell migration (Scale bar=500 µm); **(D)** Analysis of related migrated distance after treatment with MAML1 and EERAC; **(E)** Influence of MAML1 and EERAC on cell invasion (Scale bar=200 µm); **(F)** Analysis of invasive cells after treatment with MAML1 and EERAC; **(G)** Influence of MAML1 and EERAC on cell apoptosis; **(H)** Analysis of cell apoptosis after treatment with MAML1 and EERAC. *P <0.05 versus control group, # *P* <0.05 versus MAML1 group.

MAML1 has been believed to be the activator of Notch signaling pathway, and the overexpression cell model of MAML1 was established in this study. After overexpression of MAML1, the cells were denser compared with control using light microscope, but simultaneous treatment with EERAC significantly decreased cells dense (Figure 5A). Similar results were observed about the cell proliferation, migration, invasion and apoptosis. Overexpression of MAML1 remarkably promoted the proliferation, migration, and invasion, but suppressed apoptosis (Figure 5B-H). While, simultaneous treatment with EERAC and MAML1 significantly reversed the effects of MAML1. The proliferation, migration, and invasion of SW480 cells were inhibited, but cell apoptosis was increased remarkably by EERAC (Figure 5B-H). These findings further confirm the evidence that EERAC might affect colorectal cancer through targeting Notch signaling pathway.

## Discussion

CRC has become the most frequent gastrointestinal tumor in the world. In recent years, adverse side effects and drug resistance of chemotherapy has reduced success rate of CRC treatment. Nowadays, several types of plant extracts have been proved to be effective for anti-tumor with fewer side effects 23. EERAC is extracted from the radix of Actinidia chinensis, and exhibited antitumor activity. However, if EERAC could be a potential therapeutic agent for CRC and the specific mechanism remain unclear.

Notch-signaling pathway plays a vital role in influencing cell proliferation, apoptosis and differentiation 18. In recent years, overexpression of NICD1 and Jagged1 was detected in multiple types of cancer, such as CRC, prostate cancer, breast cancer and several types of lymphomas 21, 24, 25. NF-κB, DLL4, Hes-1and c-Myc are critical downstream molecules in the notch-signaling pathway, and could service as biomarkers for the recurrence, metastasis and prognosis of CRC. Therefore, notch-signaling pathway acts an important role in the occurrence and development of CRC. In this study, we elaborated the relationship between EERAC and notch- signaling pathway *in vivo* and vitro.

In this study, the growth inhibition of EERAC in CRC cancer cells depends on the dose-dependent. The cell viability of SW480 cells were decreased from 91.25% to 23.97% with various concentrations of EERAC (50-200μg/mL), respectively. In a word, EERAC has significantly inhibitive effects on the growth of SW480 cells. The invasion and migration of tumor cells are the key factors determining the malignancy of cancer. We studied the migration capacity of SW480 *in vitro*. We found that the invasion and migration capacity of SW480 cells was decreased with the increasing EERAC concentration.

The antitumor mechanism of EERAC was evaluated on SW480 cells *in vitro* by western blot. The results indicated that notch-signaling pathway downstream protein Notch1, Jagged1, and c-Myc expression were decreased in SW480 cells after treatment with EERAC suggesting that EERAC might exerted its anti-proliferative activity by targeting these downstream proteins of notch signaling pathway. In order to further confirm the suppression of EERAC on downstream proteins of notch signaling pathway, we measured the expression of c-Myc, Jagged1, and Notch1 in the tumor tissues, and similar findings were achieved. These results showed that EERAC might inhibit the levels of downstream molecules in the notch-signaling pathway to suppress the growth of tumor cells.

Our results demonstrated that EERAC had a potent inhibitory effect on the proliferation, invasion, and migration of SW480 cells. The significant suppression of EERAC on tumor growth *in vivo* was also observed. Remarkable suppression of EERAC on the downstream proteins of notch-signaling pathway (Notch1, Jagged1, and c-Myc) might be the regulatory mechanism how EERAC inhibits CRC. This study indicates that EERAC may a promising chemotherapeutic agent for CRC.

## Abbreviations

EERAC): Extracted from radix of Actinidia chinensis
CRC): colorectal cancer
TCM): Traditional Chinese Medicines
NICD): notch intracellular domain
PBS): phosphate buffer saline
FBS): fetal bovine serum
CCK-8): Cell counting kit-8
