# Ethanol Extracted from Radix of Actinidia Chinensis Inhibits Human Colon Tumor Through Inhibiting Notch-signaling Pathway: Erratum

**DOI:** 10.7150/jca.117445

**Published:** 2025-06-15

**Authors:** Wanle Hu, Chenchen Wu, Chenchen Yuan, Minyuan Chen, Chun Jin, Chenguo Zheng

**Affiliations:** Department of Coloproctology, The Second Affiliated Hospital and Yuying Children's Hospital of Wenzhou Medical University, No 109 Xueyuan Western Road, Wenzhou, Zhejiang Province, 325027, P.R. China.

In the original version of our article, the cell migration images in the figure 1 B were used from the original data of other batches of scratch experiments in this study. In addition, the protein band of Notch1 in Figure 3A and apoptosis analysis result of group 100 μg/mL in the Figure 2A are not representative images. The correct images are provided below. After check of all original data, we confirm that these corrections have no impact on the experimental outcome and conclusions. The authors apologize for any inconvenience the error may have caused. All authors agree with the erratum.

## Figures and Tables

**Figure 1:**
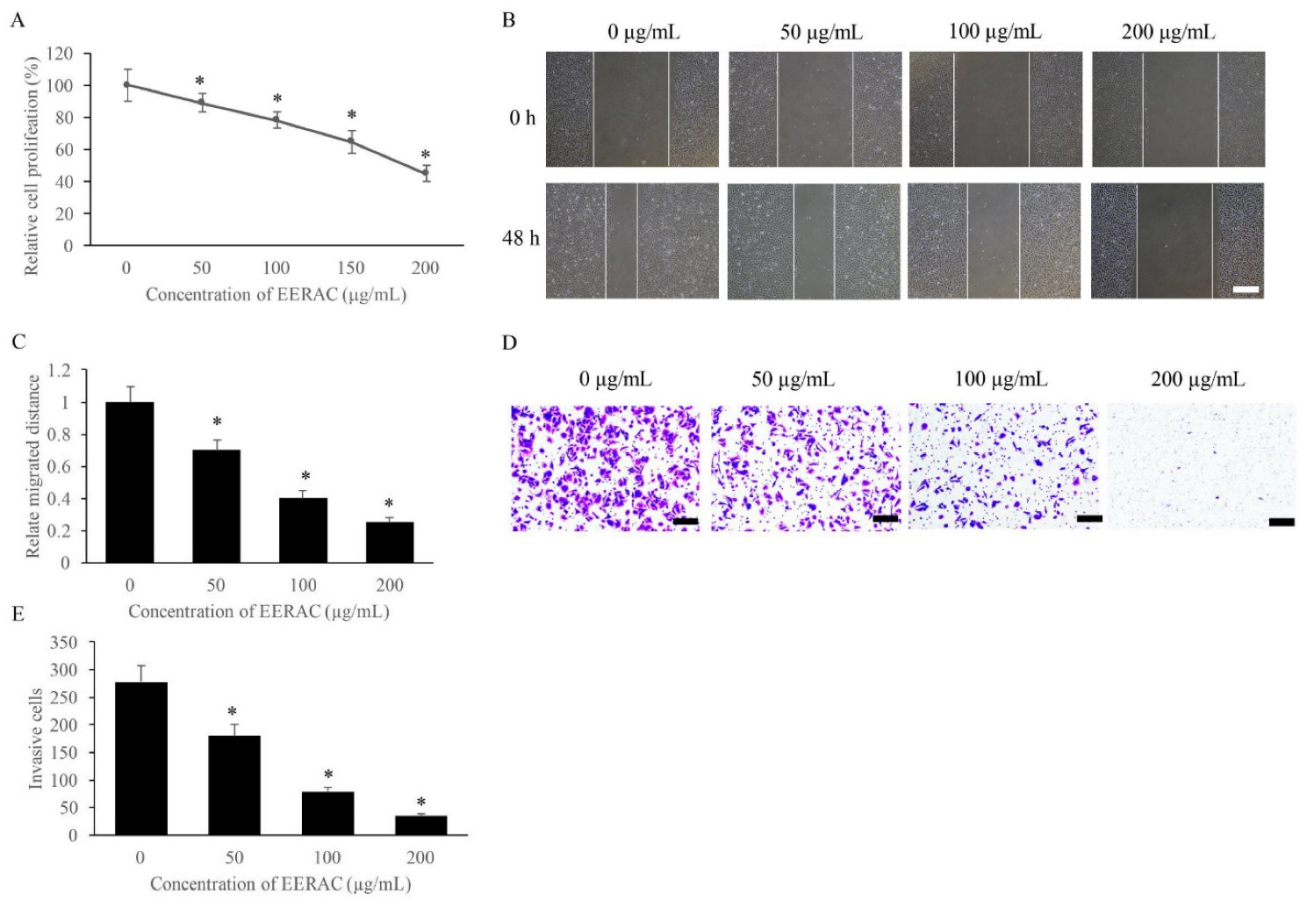
EERAC significantly inhibited the proliferation, migration, and invasion of SW480 cells. (A) Cell viability of SW480 cells treated with different concentrations of EERAC; (B) Representative images of wound healing assay after treatment with different concentrations of EERAC (Scale bar=500 μm); (C) Analysis of cell migration after treatment with different concentrations of EERAC; (D) Representative images of transwell assay after treatment with different concentrations of EERAC (Scale bar=200 μm); (E) Analysis of cell invasion after treatment with different concentrations of EERAC. *P <0.05 versus untreated EERAC (0 μg/mL).

**Figure 2:**
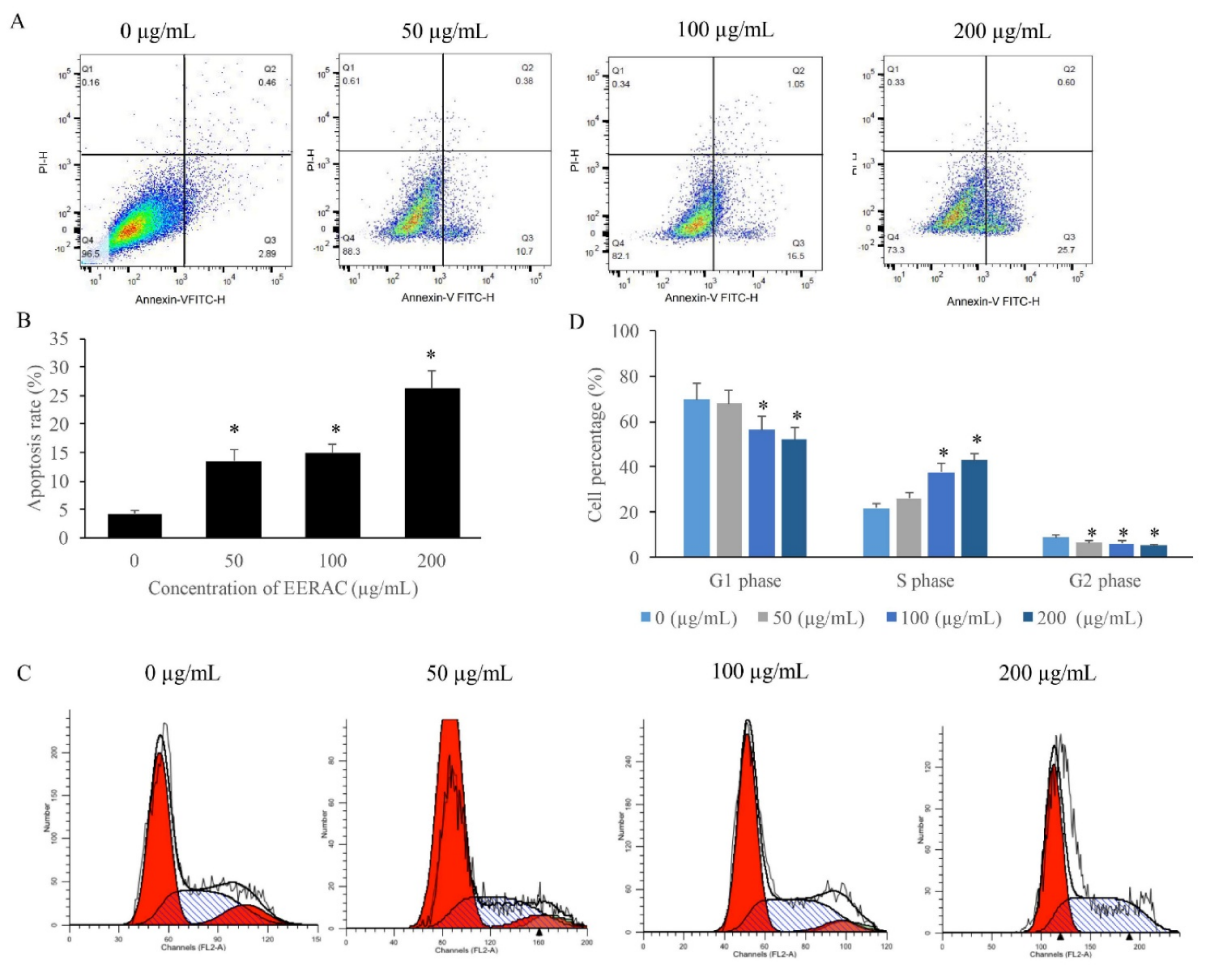
EERAC remarkably promoted the apoptosis rate of SW480 cells, and increased the cells percentage of S phase. (A) Cell apoptosis of SW480 cells was measured after treatment with EERAC; (B) Analysis of cell apoptosis after treatment with EERAC; (C) Cell cycle of SW480 cells was measured after treatment with EERAC; (D) Analysis of cell cycle after treatment with EERAC. *P <0.05 versus untreated EERAC (0 μg/mL).

**Figure 3:**
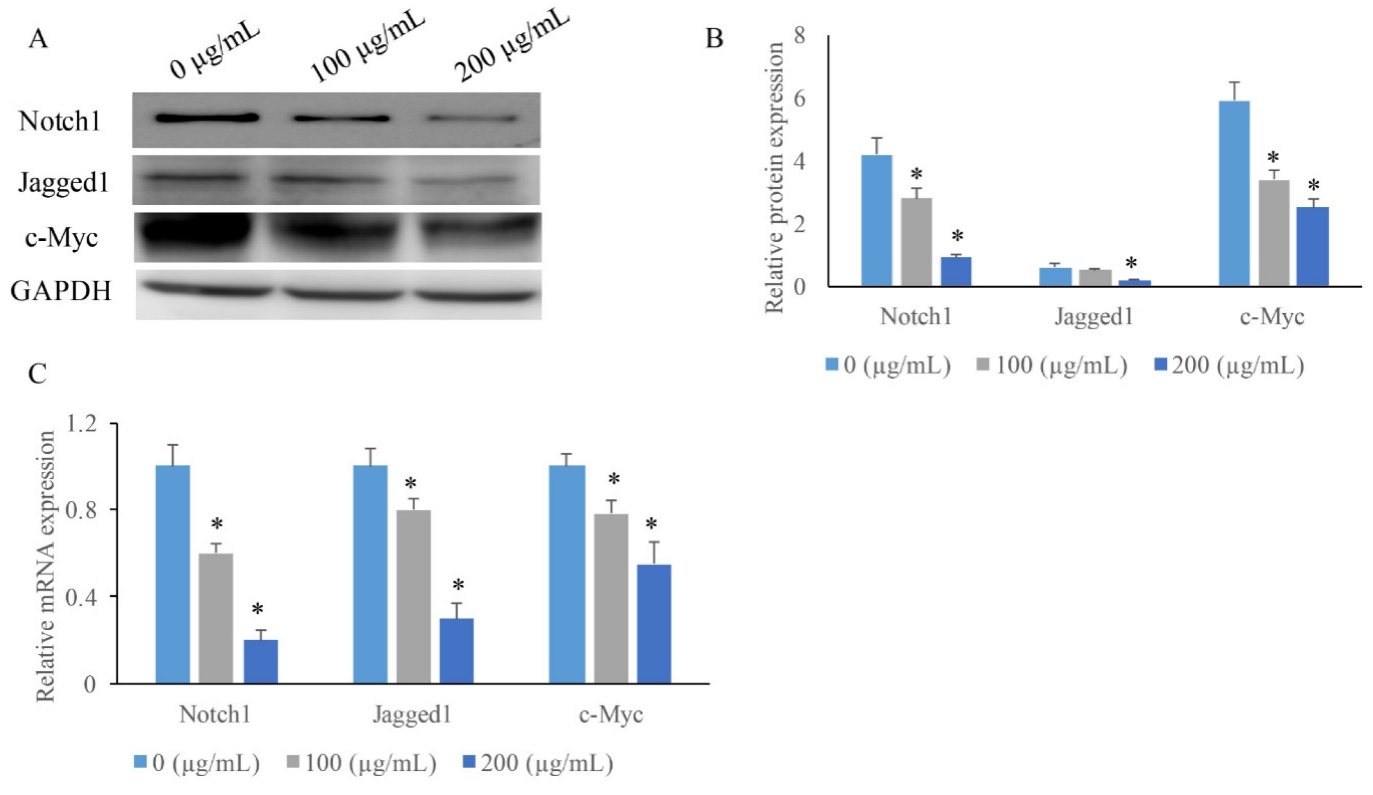
EERAC significantly inhibited the notch-signaling pathway. (A) Measurement of protein levels of c-Myc, Jagged1, and Notch1 by western blotting; (B) Analysis of protein expression of c-Myc, Jagged1, and Notch1; (C) Measurement of mRNA expression of c-Myc, Jagged1, and Notch1 by qRT-PCR. *P <0.05 versus untreated EERAC (0 μg/mL).

